# Detection of Pseudorabies Virus in Wild Boar Foetus

**DOI:** 10.3390/ani10020366

**Published:** 2020-02-24

**Authors:** Maria Irene Pacini, Mario Forzan, Giovanni Cilia, Lucrezia Bernardini, Filippo Marzoli, Francesca Pedonese, Patrizia Bandecchi, Filippo Fratini, Maurizio Mazzei

**Affiliations:** Department of Veterinary Sciences, University of Pisa, Viale delle Piagge 2, 56124 Pisa, Italy; mariairene.pacini@phd.unipi.it (M.I.P.); mario.forzan@unipi.it (M.F.); giovanni.cilia@vet.unipi.it (G.C.); lucrezia.bernardini@gmail.com (L.B.); filippo.marzoli@gmail.com (F.M.); francesca.pedonese@unipi.it (F.P.); patrizia.bandecchi@unipi.it (P.B.); filippo.fratini@unipi.it (F.F.)

**Keywords:** pseudorabies virus, wild boar, Italy, foetus, Suid alphaherpesvirus 1, transmission route

## Abstract

**Simple Summary:**

Pseudorabies virus (PRV) is a worldwide pathogen primarily affecting domestic and wild swine. In wild boar, seroprevalence rates are high, but little information is available about the impact of the disease on reproduction in this wild species. Our aim s to investigate the presence of Pseudorabies virus in foetus samples collected from pregnant animals living in an endemic area. A polymerase chain reaction (PCR) positive foetus sample is identified from a seropositive and viral shedding sow. Sequence analysis confirms the molecular result, describing for the first time the viral presence in wild boar foetus, suggesting an additional transmission route.

**Abstract:**

Pseudorabies, or Aujeszky’s disease, is a notifiable worldwide infection of domestic and feral swine that causes economic losses for the swine industry. In domestic pigs, the virus is responsible for nervous and/or respiratory symptoms; in pregnant sows, it is one of the major causes of stillbirth, mummification, embryonic death, and infertility (SMEDI). It is known that PRV infection in wild boar is associated with low pathogenicity and attenuated or absent symptomatology, but limited information is available about the ability of the virus to infect the foetuses of infected wild boar pregnant sows. Due to scarce information about the reproductive consequences, we investigate the possible intrauterine vertical transmission of the virus in wild boar pregnant sow living in a highly infected area. A number of 54 hunted wild boar were sampled during 2018–2019, and blood, genital and nasal swabs, placenta, and fetuses were collected for serological and molecular investigations. A seroprevalence of 74% (40/54) was detected, while 1/24 pregnant sow and 1/24 pooled foetuses tested positive by PCR (gene gB). This is the first evidence of viral detection in foetuses from seropositive pregnant wild boar. This finding suggests the possible pathogenetic role of PRV on pregnancy in wild boar and the existence of an additional transmission route.

## 1. Introduction

Pseudorabies (PR) or Aujeszky’s disease is a notifiable worldwide infection of domestic and feral and wild swine that was first described in the early twentieth century [1,2]. The disease is characterised by neurological and reproductive porcine disorders causing economic losses for the swine industry.

The causative agent is Suid herpesvirus 1 (SuHV-1), also known as pseudorabies virus (PRV), or Aujeszky’s disease virus (ADV), a member of the family Herpesviridae, subfamily Alphaherpesvirinae, genus Varicellovirus [2].

PRV can infect many species, but despite its wide host range, which includes nearly all mammals, the natural hosts for PRV are members of the Suidae family, in particular domestic pigs and wild boar. In these animals, the virus is able to establish a lifelong latent infection in neuronal and non-neuronal cells, so swine and wild boar survive the infection and behave like a reservoir of PRV [3,4].

In pigs, PRV transmission mainly occurs by an oro-nasal route due to the high density of swine in farms, which allows for nose-to-nose contact and disease shedding. The venereal transmission has been suggested as the main route in feral swine and wild boar [2,5,6]. Higher viral seroprevalence in females, along with the presence of viral DNA in nasal secretions, suggests that PRV is mainly transmitted oro-nasally within female groups throughout the year, whereas venereal transmission is limited to the mating season [3,6,7]. Secondary routes of transmission are through contact with fomites or by ingestion of contaminated carcasses of other infected animals, such as feral swine, wild boar, rodents, or carnivores [2,8,9].

Due to great economic impact on the swine industry, most of the European countries have implemented eradication programs with the purpose of eradicating PRV and guaranteeing free trade within Europe [10]).

Since the 1980s, PR has spread globally due to the changes in swine management with the rise of intensive farming and to the appearance of highly virulent strains of PRV [2,11]. Therefore, infection, prevention, and control plans including large-scale vaccination with gE-deleted vaccines have been put in place in farmed pigs. To date, the disease has been eradicated within the domestic pig population in several European nations such as Denmark, Finland, Austria, France, Germany, Hungary, Switzerland, Sweden, Slovakia [2]. Canada, New Zealand, and the United States have been declared as “Aujeszky’s Disease-free” [12,13].

On the other hand, in countries where domestic pigs are PRV free, the virus is almost always present in an endemic form in wild boar [10]. In fact, the PRV seroprevalence in wild boar in European countries ranges from 4% to 66%, and therefore it is important to understand the epidemiology of the virus in this species in order to avoid the possibility of relapses in pigs or infection of other susceptible animals [2,6,10,14,15,16,17,18,19,20,21,22].

To gain information concerning the epidemiology of the virus, genetic characterisation of circulating PRV genotypes can be helpful.

PRVs are classified by the Restriction Fragment Length Polymorphism (RFLP) analysis in four major types and several subtypes [4]: genotype I, found mainly in the USA and in Central Europe; genotypes II and III, circulating in Northern Europe and Central Europe; and genotype IV, which is limited to Asia [2,23].

In Europe, while genotype 1 mainly circulates in wild boar, both genotype 1 and 2 have been detected in domestic pigs even if the latter is much more widespread [20,23].

In Italy, although the National Authority has implemented an eradicating program adopting severe and restrictive measures, the virus has undergone a substantial reduction in circulation in pig farms but has not yet been eradicated; furthermore, it is widely spread within the wild boar population [10,24].

In adult domestic pigs, the virus is responsible for respiratory symptoms of different severities that can determine the worsening of the general health condition, loss of weight and appetite, and therefore a decrease in production performance. While in adult pigs morbidity is very high and mortality is around 1%–2%, in piglets, there are nervous symptoms, including tremors, convulsions, and paralysis, leading to death in 100% of cases. In pregnant sows, the infection, as well as the reactivation of the virus, leads to stillbirth, mummification, embryonic death, and infertility (SMEDI) with embryo resorption, foetal mummification, abortion, or stillbirth based on the month in which the virus reaches the placenta [25,26,27,28,29].

Unfortunately, little is known about the symptomatology of the disease in wild boar. So far, it is known that PRV infection in free-ranging wild boar is associated with low pathogenicity and attenuated or absent symptomatology with only mild respiratory symptoms. Limited information is available about the ability of infected wild boar pregnant sows to carry out the pregnancy [2,15,17,30].

Due to scarce information about the reproductive consequences of PRV infection, we investigate the implications of the virus in wild boar pregnant sow living in a highly infected PRV area.

## 2. Materials and Methods

### 2.1. Sample Collection

During the 2018–2019 hunting seasons (from November to January), 54 adult (>24 months) wild boar hunted in Tuscany, following Regional Hunting Law (Regolamento di attuazione della legge regionale 12 gennaio 1994 n° 3 DPGR 48/R/2017) in an area characterised by the abundant presence of wild boar and other wild ungulates [31] (Pisa, Siena, Grosseto, and Livorno province), were sampled. The territorial contiguity of the sampling sites allows one to consider all the sampled wild boar as belonging to a single epidemiological population. Animals were classified by sex in 20 males and 34 females, of which 24 were pregnant. During the post-mortem operation, serum was collected from all animals from the infraorbital cavity [32]. In addition, nasal and genital swabs were taken from all pregnant sows. Finally, pregnant uteruses (*n* = 24) were sampled and transported to the Department of Veterinary Science (University of Pisa), where placenta and foetuses were collected and stored at −20 °C until molecular analysis.

### 2.2. Serologic Analysis

Serum samples were analysed by ID Screen Aujeszky gB competitive kit detecting anti-gB PRV antibodies (ID.vet, Grabels, France). Test procedures and interpretation of results were performed according to the manufacturer’s instructions adopting the short serum incubation protocol. The optical density was measured by a plate reader (Multiscan FC; Thermo Scientific, Waltham, MA, USA) at 450 nm wavelength.

### 2.3. Molecular Analysis

The DNA extraction was performed on placenta samples and pooled tissues of the foetuses collected from each pregnant sow using the DNeasy Blood and Tissue kit (Qiagen, Hilden, Germany) following the manufacturer’s instruction. DNA was eluted in 70 µL Buffer AE and stored at −20°C until analysis. Nasal and genital swabs were thawed and soaked in DNase free water, then vortexed for 30 seconds and centrifuged. The supernatants collected from nasal and genital swabs were pooled and used as a single PCR template. The PCR assay was performed on all placenta, pooled foetuses, and swabs to identify the *ul27* gene (gB) the most highly conserved glycoproteins [33,34], using Wonder Taq DNA polymerase (Euroclone, Milano Italy). 

### 2.4. Phylogenetic Analysis

Nucleotide sequence analysis was performed on PCR positive samples derived from foetal tissues (BMR Genomics, Padova, Italy) in order to confirm the specificity of PCR assay. A panel of PRV gB GenBank-available sequences was selected to perform phylogenetical analysis by maximum-likelihood methods as available in the MEGA6 software package [35]. A phylogenetic tree was then generated. 

### 2.5. Statistical Analyses

Data were analysed by Chi-square (*χ*^2^) tests to evaluate the difference in the seropositive rate between males and females and between pregnant and nonpregnant females. The statistical significance threshold was set at *p*-value ≤ 0,05. 

## 3. Results

### 3.1. Serologic Analysis

The serological analysis indicated that 40 out of 54 sera (74%) scored positive for anti-gB PRV antibodies. Of the positive samples, 14/20 (70%) belonged to males and 26/34 (77%) belonged to female wild boar. In addition, 19 out of 24 pregnant sows (79%) scored positive (Table 1).

### 3.2. Molecular Analysis

The PCR assay for the detection of gB gene identified one positive pooled foetuses’ sample and one positive swab; no positives were identified in any of the placenta tissues examined. The positive pooled foetuses and the positive swab that resulted were collected from the same pregnant sow. 

### 3.3. Phylogenetic Analysis

**Figure 1:**
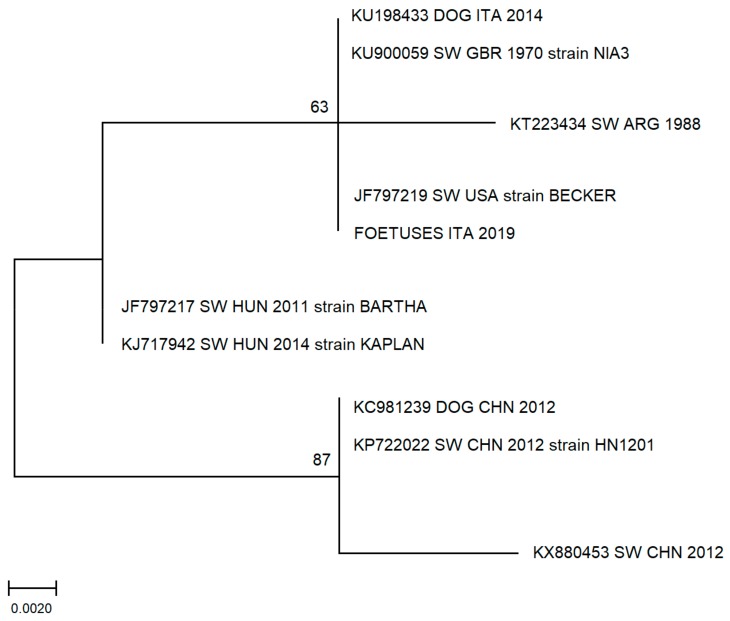
Molecular phylogenetic analysis by maximum likelihood method. The evolutionary history was inferred by using the maximum likelihood method based on the Tamura–Nei model. The percentage of replicate trees in which the associated taxa clustered together in the bootstrap test (500 replicates) is shown next to the branches. The analysis involved 10 nucleotide sequences. There was a total of 148 positions in the final dataset. Evolutionary analyses were conducted in MEGA7. (ITA: Italy; GBR: Great Britain; ARG: Argentina; USA: United States of America HUN: Hungary; CHN: China; SW: swine).

Sequence analysis confirmed the specificity of PCR product, and a phylogenetic tree shows high homology to other sequences derived from Italian or European suids (Figure 1). In particular, the sequence obtained in this study shows high homology to a PRV sequence obtained in 2014 from a hunting-dog (KU198433 DOG ITA 2014) living in the province of L’Aquila, Abruzzi region, Central Italy that came in contact with the blood of a wild boar [36].

### 3.4. Statistical Analyses

No statistical difference (*p* > 0,05) has been evidenced on seropositive rates between males and females, neither between pregnant nor nonpregnant females.

## 4. Discussion

Our study indicates a PRV antibody prevalence in adult wild boar of 74% in accordance with previous studies describing that the infection is endemic in wild boar populations in the study area and supporting the data reported for adult wild boar in other works carried out in Central and Southern Italy [6,24,37].

On the other hand, our seroprevalence value is clearly higher than those found in Liguria [38] and along the Alps [15,39].

This difference is due to the different population densities of wild boars between two distinct geographical areas: in the Alps, in Northern Italy, the population density is low, while in Central-Southern Italy the number of wild boars is much higher [15]. Antibody prevalence is also similar to those previously reported in most parts of Europe [2,3,14,16,22].

No differences in seroprevalence between the two sexes were found in this study. This appears to be in contrast with the previous literature, in which higher seroprevalence rates are generally reported in females. In fact, sows live in “maternal groups” [3,9,39] and therefore have a greater possibility of oronasal transmission of the virus between individuals, compared to males who are solitary and meet their conspecifics only during the mating season. 

This result can be due to the high density of wild boar population and the high level of PRV seroprevalence in the study area [6,31], which are factors that could even out sex differences in seroprevalence. Moreover, the low number of samples analysed did not permit a complete epidemiological investigation.

Considering the virological analysis, only one out of 24 pregnant sows shed the virus, as indicated by the PCR positive result on the nasal-genital swab. This data is in accordance with other studies that identified percentages of shedding individuals ranging from 1% to 6% inside of a population with high seroprevalence rates (50%–60%) [7,9]. Interestingly, pooled foetuses collected from the pregnant sows also resulted in positive to virological analysis, indicating the ability of PRV to infect the foetuses during pregnancy in a viraemic status. Unfortunately, the exact number of infected foetuses is unknown since we analysed pooled samples. Nonetheless, the data are sufficient to demonstrate that even in a wild species the PRV virus has a potential reproductive impact. No gross lesions, no signs of abortion or foetal mummification, and no differences in foetal size were reported at the time of the samples collection.

Although the swabs and the foetuses tested positive, the placenta of this shedding sow was negative, as was that of all the other pregnant females. This result is not unexpected, since other authors have already described the same feature in infected pregnant domestic sows [30]. In addition, we had the possibility to collect only a small portion of the placenta due to the complexity of sampling.

Since the aim of the research was to identify the majority of the PRV circulating in the study area, the PCR assay was based on gB glycoprotein, the most conserved portion of the genome [34]. The sequence analysis of the virological positive sample showed high homology to other European sequences and in particular with a sequence retrieved from a hunting dog in Italy living in a nearby district [36]. 

According to numerous studies, two distinct cycles of pseudorabies virus are described, one involving wild boar with mild or nonexistent symptoms and the other involving domestic pigs. The two cycles are apparently quite separate, and the threat of transmission of the virus among wild boar and domestic suids is rather limited. More recent works, however, underline that the passage of the PRV among the two species cannot be excluded and that therefore it is necessary to be cautious and supervise all the possible ways of contagion [2,10,20,40].

Unfortunately, the quality of the sample and its extracted DNA did not allow us to carry out more accurate phylogenetic investigations through PCR on variable genes. Therefore, it was not possible to establish whether the detected virus is genetically related to the type of PRV circulating in wild boar populations or to that circulating on commercial farms.

This data is a further confirmation of active circulation of the PRV in the wild boar population, and confirms that they represent a source of infection for other wild or domestic species, resulting in new possible epidemiological scenarios. Nevertheless, no clinical signs have been reported from hunters or forest rangers in our sampled animals.

## 5. Conclusions

In conclusion, this work is the first evidence of viral detection in foetuses collected from seropositive pregnant wild boar living in an endemic area in Italy. The findings suggest the possible pathogenetic role of PRV on pregnancy in wild boar.

This data could be useful to further studies in order to deeply investigate the capacity of the virus to determine a SMEDI effect as described in domestic swine. This possibility could also increase transmission routes, not only among wild boar but also to other wild or domestic animals that could have contact with infected aborted foetuses.

## Figures and Tables

**Table 1:** Enzyme-linked immunosorbent assay (ELISA) serological results and seroprevalences percentage of sampled wild boar grouped by sex and pregnancy/no pregnancy status.

	SEROPOSITIVES		SERONEGATIVES	
**MALES**	14/20 (70%)		6/20 (30%)	
**FEMALES**	26/34 (77%)		8/34 (23%)	
	*PREGNANT* 19/24 (79%)	*NO PREGNANT* 7/10 (70%)	*PREGNANT* 5/24 (21%)	*NO PREGNANT* 3/10 (30%)
**TOT**	40/54 (74%)		14/54 (26%)	
